# Improving Adherence to Perioperative Cognitive Assessment Documentation in Hip Fracture Patients: A Quality Improvement Project

**DOI:** 10.7759/cureus.88482

**Published:** 2025-07-21

**Authors:** Nicholas Tse Hao Ng, Sally Li Er Fong

**Affiliations:** 1 Trauma and Orthopedics, King's Mill Hospital, Sherwood Forest Hospitals NHS Foundation Trust, Sutton-in-Ashfield, GBR

**Keywords:** 4at, abbreviated mental test score (amts), clinical documentation improvement, clinical practice guideline, cognitive assessment, fragility hip fracture, hip fracture management, post-operative delirium, quality improvement (qi), trauma and orthopedic surgery

## Abstract

Background

Perioperative cognitive assessments are recommended by the National Institute for Health and Care Excellence (NICE) for all hip fracture patients as part of best practice tariffs. The Abbreviated Mental Test Score (AMTS) and 4A's Test (4AT) are widely used tools for cognitive assessments. This quality improvement project aimed to assess and improve adherence to AMTS and 4AT documentation protocols in hip fracture patients within our department.

Method

Two prospective audits were conducted on all inpatients with hip fractures in our department between April and July 2024. Data on patient demographics, adherence to AMTS and 4AT documentation protocols, and the presence of postoperative delirium were collected. Admission AMTS was defined as the documentation of AMTS within 24 hours of the patient's presentation to the emergency department. Educational posters were displayed in clinical areas as part of a targeted intervention between each audit cycle.

Results

A total of 49 patients were identified across both cycles, with 19 in the first cycle and 30 in the second. About 15 (78.9%) patients had an admission AMTS documented by the admitting doctor or orthogeriatric team. About 18 (94.7%) patients had a documented postoperative AMTS, but only nine (47.4%) had a postoperative 4AT documented. No patients developed postoperative delirium during the first cycle. Following our educational initiatives, 29 (96.7%) patients had a documented admission AMTS. Postoperatively, AMTS and 4AT documentation were present in 26 (86.7%) and 18 (60.0%) patients, respectively. Six (20.0%) patients developed postoperative delirium in the second cycle.

Conclusion

This quality improvement project highlights the importance of staff education in improving adherence to documentation. However, complete adherence has not yet been achieved. The presence of postoperative delirium in a significant number of patients emphasizes the importance of prompt cognitive assessment to improve patient outcomes in this vulnerable cohort. Further efforts should identify barriers to the consistent assessment and documentation of AMTS and 4AT scores.

## Introduction

Hip fractures are one of the most common injuries in older adults, often leading to significant morbidity, mortality, and prolonged hospital stays. Perioperative cognitive impairment is common in this vulnerable cohort, with up to 50% of hip fracture patients experiencing delirium during hospitalization [1]. Early detection of cognitive decline, including the risk of delirium, is vital to tailoring clinical management to improve outcomes [2]. The Abbreviated Mental Test Score (AMTS) and the 4A's Test (4AT) are widely recognized tools and are recommended for assessing cognitive function in patients at risk of delirium [3].

The National Institute for Health and Care Excellence (NICE) guidelines recommend that all patients aged 65 and over with a hip fracture undergo an early cognitive assessment using validated tools, such as the AMTS or 4AT, within 24 hours of hospital admission. Regular cognitive assessments should be conducted to identify patients at risk of postoperative delirium and guide interventions aimed at reducing its incidence [4]. However, incomplete or delayed documentation of these assessments can hinder clinicians' ability to effectively manage these vulnerable patients.

Despite these recommendations, adherence to cognitive assessment documentation protocols remains inconsistent within clinical practice [5]. Studies have shown that under-documentation of cognitive scores, including the AMTS and 4AT, is common and often results from time constraints, lack of awareness, or the low prioritization of cognitive evaluations. Incomplete documentation hinders early intervention in patients at risk of cognitive decline and postoperative delirium [6]. Therefore, our quality improvement project aims to evaluate and improve adherence to perioperative cognitive assessment documentation in hip fracture patients, in accordance with national guidance.

## Materials and methods

**Figure 1:**
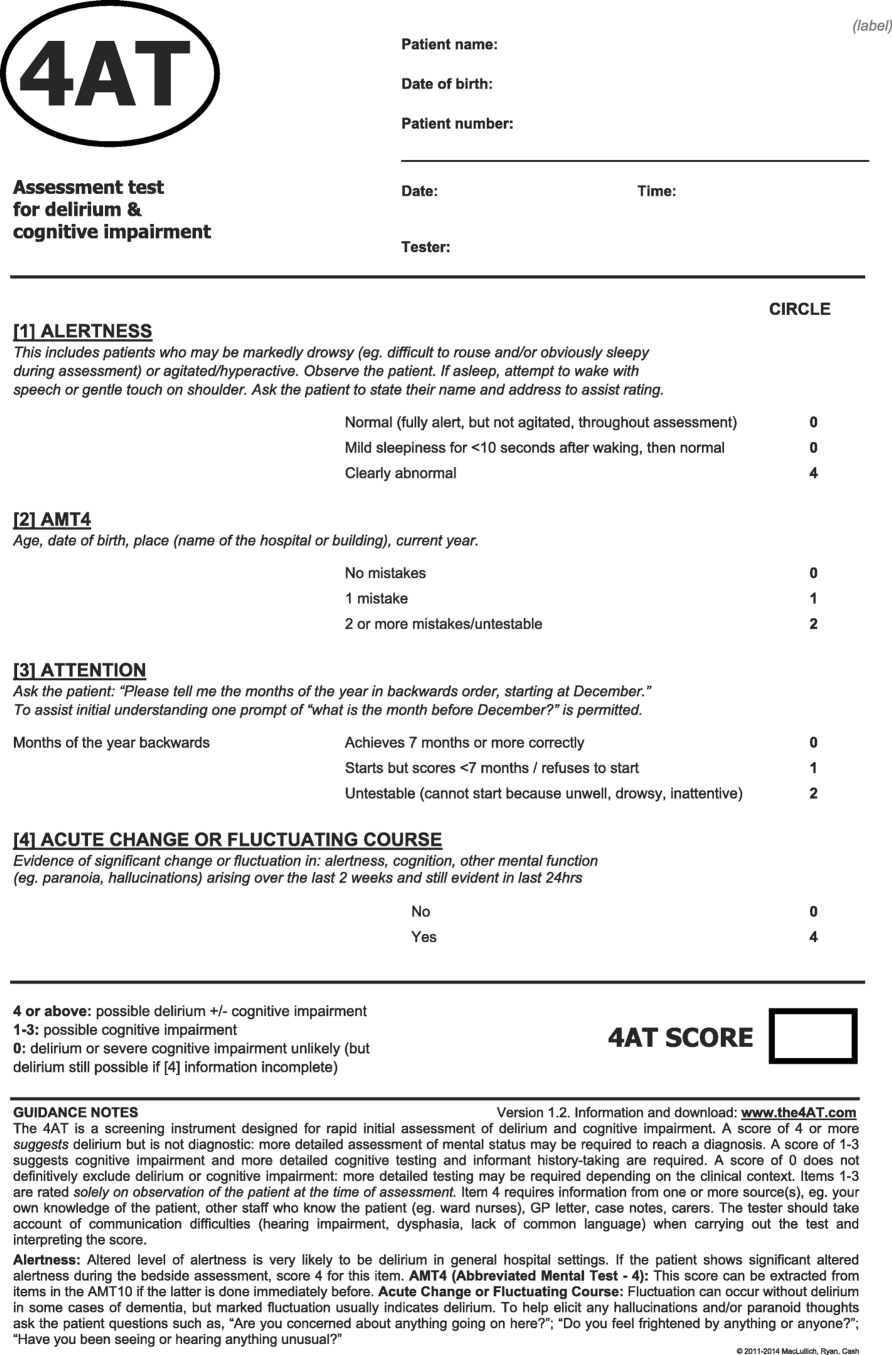
The 4A's Test (4AT) Adapted from the 4AT website. https://www.the4at.com.

Two prospective audits were conducted in our department in April and July 2024. All inpatients aged 65 years and older with hip fractures were included. An age cut-off of 65 years was established to align with the patient cohort targeted by the NICE standards being audited. The primary outcome of the audits was to evaluate adherence to the documentation protocols for the Abbreviated Mental Test Score (AMTS) (Table 1) and the 4A's Test (4AT) (Figure 1).

**Table 1:** Abbreviated Mental Test Score (AMTS). Adapted from the British Geriatric Society website. https://www.bgs.org.uk/sites/default/files/content/attachment/2018-07-05/abbreviated_mental_test_score.pdf.

Questions	Score
What is your age?	
What is the time to the nearest hour?	
Give the patient an address, and ask him or her to repeat it at the end of the test, e.g., 42 West Street	
What is the year?	
What is the name of the hospital or number of the residence where the patient is situated?	
Can the patient recognize two persons (the doctor, nurse, home help, etc.)?	
What is your date of birth? (day and month sufficient)	
In what year did World War 1 begin?	
Name the present monarch/prime minister/president	
Count backwards from 20 down to one	
Total score	

Data collection for the initial cycle spanned one week (22-28 April 2024), while the subsequent cycle was extended to two weeks (1-14 July 2024). The initial one-week data collection period was sufficient to identify a gap in 4AT documentation, prompting the need for early intervention. The second cycle was extended to two weeks to increase the sample size, thereby improving the robustness of the analysis and any observed changes were more representative of the cohort.

Data were collected through a review of medical records, radiographs, patient demographics, adherence rates for the AMTS and 4AT documentation, and the incidence of postoperative delirium. The admission AMTS was defined as any documented AMTS score recorded within 24 hours of the patient's arrival at the emergency department. Admission and postoperative day one assessments were performed by orthopedic resident doctors, ranging from FY2 (postgraduate training year two) to CT2 (postgraduate training year four), as well as orthogeriatric registrars and consultants. Postoperative 4AT was routinely performed by orthopedic resident doctors ranging from FY2 to CT2.

**Figure 2:**
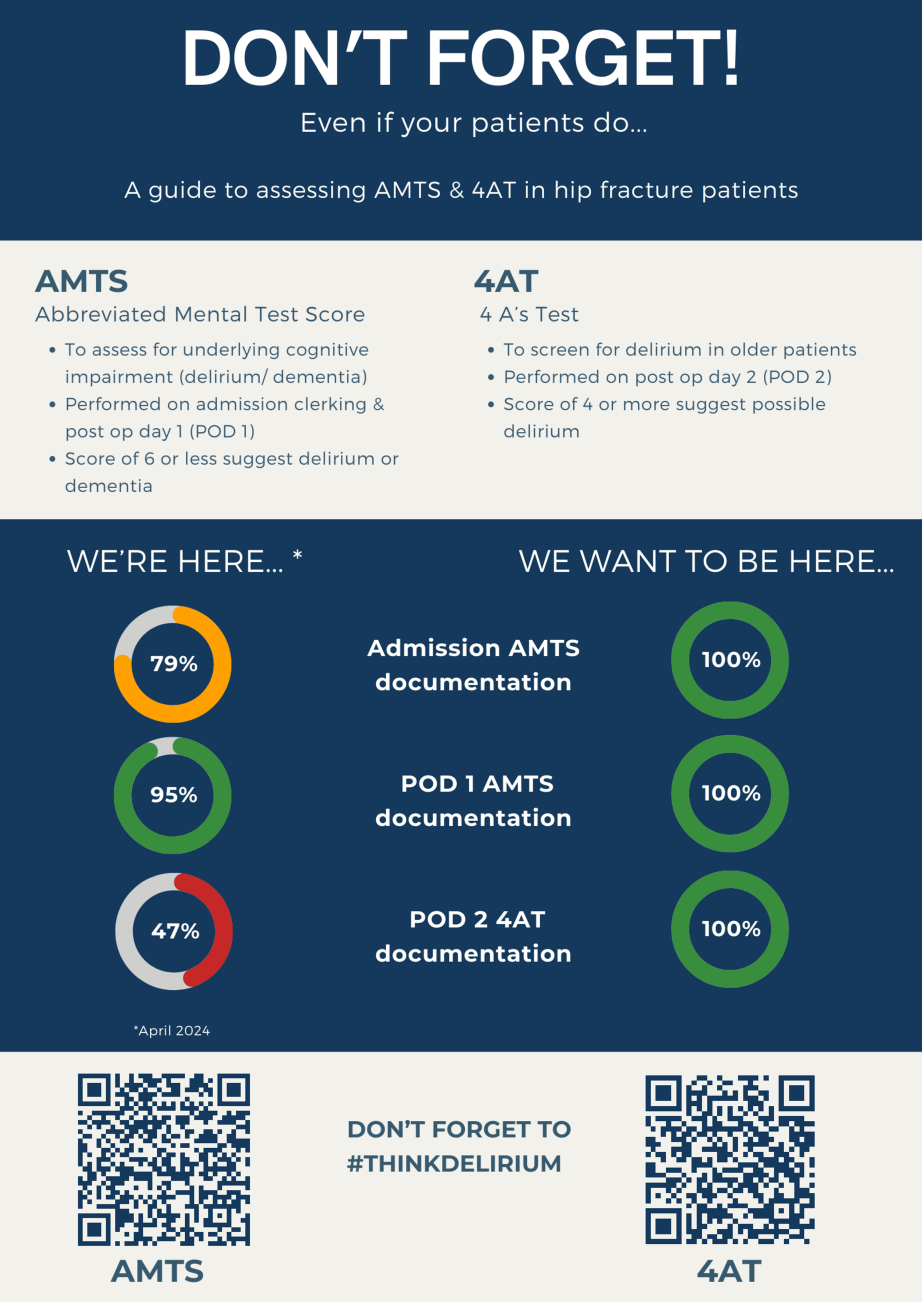
Educational poster. Adapted from Sherwood Forest Hospitals (SFH) NHS Foundation Trust, educational posters with QR codes displayed on wards and doctors' office, with permission. Designed by Nicholas Ng (Core Surgical Trainee, Trauma and Orthopedics, Kings Mill Hospital, SFH NHS Foundation Trust).

Between the two audit cycles, a targeted educational intervention was implemented. This included displaying educational posters (Figure 2) with quick response (QR) codes for AMTS and 4AT calculators to enhance accessibility and staff training sessions designed to raise awareness about the importance of cognitive assessments and proper documentation. Following the second audit cycle, the data were analyzed to assess changes in adherence rates to documentation protocols, allowing for the identification of barriers and facilitators related to the documentation process. This approach aimed to enhance cognitive assessment documentation in the management of hip fracture patients.

## Results

A total of 49 patients with hip fractures were included across both audit cycles, with 19 patients in the first cycle and 30 patients in the second cycle. In the first audit cycle, 15 (78.9%) patients had an AMTS documented within 24 hours of admission, either by the admitting physician or the orthogeriatric team. There was no difference in documentation of admission assessments between admitting physicians and the orthogeriatric team. Postoperatively, the adherence to AMTS documentation was better, with 18 (94.7%) patients having a postoperative AMTS recorded. However, the documentation rate for the 4AT was lower, with only nine (47.4%) patients having a postoperative 4AT assessment completed. Importantly, no cases of postoperative delirium were identified in this first cycle.

After the implementation of educational interventions, which included posters and a training session for doctors in the department, documentation rates improved in the second audit cycle. These interventions were introduced a week after the initial cycle, and the second cycle of data was collected two months after the intervention. Of the 30 patients assessed in this cycle, 29 (96.7%) had an admission AMTS documented. Postoperative AMTS documentation remained high, with 26 (86.7%) patients having a documented score. Documentation of the 4AT also improved, with 18 (60.0%) patients having a postoperative 4AT assessment recorded.

**Figure 3:**
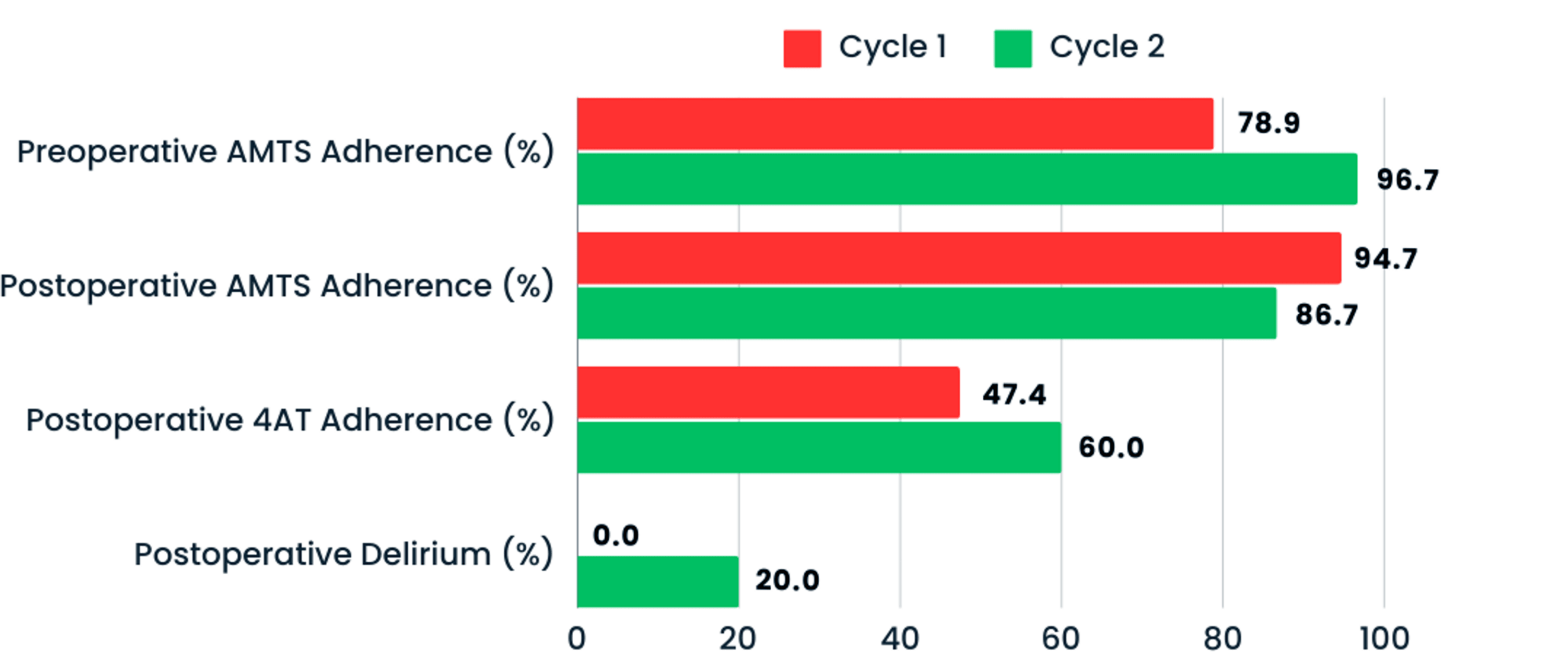
Perioperative AMTS and 4AT documentation adherence and postoperative delirium incidence. Adherence to pre- and postoperative AMTS documentation, postoperative 4AT documentation, and the incidence of postoperative delirium between both cycles. AMTS: Abbreviated Mental Test Score; 4AT: 4A's Test.

Despite the improvement in documentation adherence, postoperative delirium was identified in six (20.0%) patients in the second cycle. This represents a notable increase in delirium incidence compared to the first cycle (Figure 3).

## Discussion

This quality improvement project demonstrated improvements in the documentation of perioperative cognitive assessments, specifically the AMTS and the 4AT, in hip fracture patients following targeted educational interventions. Our results revealed an increase in admission AMTS documentation from the first audit cycle to the second cycle. Additionally, postoperative AMTS documentation remained high across both audit cycles. These findings suggest that educational posters and staff training sessions were effective in raising awareness about the importance of early cognitive assessments. This supports the results of a study by Fong et al., which demonstrated that targeted educational interventions can significantly improve adherence to clinical protocols [6].

Although the 4AT documentation rate improved between cycles, full compliance has yet to be achieved. This suggests that while educational initiatives are effective, they may not be enough to address all barriers to documentation. Potential reasons for incomplete adherence to cognitive assessments could include time constraints and competing clinical priorities [5]. Assessing cognitive function in elderly patients who may have underlying cognitive impairment can be challenging and can further complicate adherence to standardized documentation protocols [2]. Further efforts are required to identify barriers to achieving better adherence to 4AT documentation.

While no differences were observed in the documentation of admission and postoperative day-one AMTS between orthopedic resident doctors and orthogeriatric registrars or consultants, undocumented assessments may reflect weekend admissions when orthogeriatric cover was unavailable, although the difference in weekday and weekend admissions was not specifically examined in this audit. The lower rate of postoperative day-two 4AT documentation could be attributed to its routine completion by resident doctors, with orthogeriatric review only occurring if specific concerns were identified.

The higher incidence of postoperative delirium, despite improved documentation of cognitive assessments, may reflect greater adherence to documentation protocols, leading to more accurate preoperative baseline assessments and postoperative comparisons. Thus, highlighting the importance of these assessments for early recognition of this condition. Conversely, this finding might also indicate that while documentation has improved, actions to mitigate it may not have been consistent, leading to the observed increase in delirium cases. Therefore, it is paramount that clinical actions be taken when delirium is identified, as good documentation alone is not sufficient. Early cognitive assessment allows for the implementation of strategies, such as minimizing polypharmacy, promoting early mobilization, and ensuring adequate hydration, which are known to reduce the risk of delirium [3]. 

This study had several limitations, including a small cohort size, a short study period, the lack of subgroup analysis on patients with pre-existing cognitive impairment, selection, and observer bias. The small total sample size of 49 patients across both cycles limits the ability to draw statistically significant conclusions from the results. The short study period may not reflect the documentation practices of all doctors in the department, as only a subset of clinicians was observed. The outcomes may also reflect the natural development in resident doctor competency throughout the rotation. Subgroup analysis of patients with pre-existing cognitive impairment would provide a more robust analysis. Additionally, selection and observer bias may be present, as the cohort may not represent the general population of hip fracture patients, and there was no blinding of the outcomes. Future studies should include a larger sample size and longer study timeframes to incorporate more diverse patient and clinician groups to produce more generalizable and statistically robust findings.

## Conclusions

In conclusion, this two-cycle quality improvement project demonstrates that educational interventions can improve the documentation of cognitive assessments in hip fracture patients. However, more comprehensive strategies are required to improve adherence to cognitive assessments in efforts to reduce the incidence of postoperative delirium. Identifying barriers to the consistent use of both the AMTS and 4AT scores is crucial, as it allows targeted interventions to be implemented to support long-term adherence to best practice guidelines for cognitive assessments in hip fracture patients.
